# Diagnosing sexually transmitted infections in resource‐constrained settings: challenges and ways forward

**DOI:** 10.1002/jia2.25343

**Published:** 2019-08-30

**Authors:** Teodora EC Wi, Francis J Ndowa, Cecilia Ferreyra, Cassandra Kelly‐Cirino, Melanie M Taylor, Igor Toskin, James Kiarie, Nancy Santesso, Magnus Unemo

**Affiliations:** ^1^ Department of Reproductive Health and Research World Health Organization Geneva Switzerland; ^2^ Skin & Genito‐Urinary Medicine Clinic Harare Zimbabwe; ^3^ Department of Emerging Threat and AMR FIND Geneva Switzerland; ^4^ Department of Clinical Epidemiology and Biostatistics McMaster University Ontario Canada; ^5^ World Health Organization Collaborating Centre for Gonorrhoea and other STIs Department of Laboratory Medicine Faculty of Medicine and Health Örebro University Örebro Sweden

**Keywords:** STD/STI, point of care, diagnostics, key and vulnerable populations, treatment

## Abstract

**Introduction:**

Sexually transmitted infections (STIs) remain prevalent and are increasing in several populations. Appropriate STI diagnosis is crucial to prevent the transmission and sequelae of untreated infection. We reviewed the diagnostic accuracy of syndromic case management and existing point‐of‐care tests (POCTs), including those in the pipeline, to diagnose STIs in resource‐constrained settings.

**Methods:**

We prioritized updating the systematic review and meta‐analysis of the diagnostic accuracy of vaginal discharge from 2001 to 2015 to include studies until 2018. We calculated the absolute effects of different vaginal flowcharts and the diagnostic performance of POCTs on important outcomes. We searched the peer‐reviewed literature for previously conducted systematic reviews and articles from 1990 to 2018 on the diagnostic accuracy of syndromic management of vaginal and urethral discharge, genital ulcer and anorectal infections. We conducted literature reviews from 2000 to 2018 on the existing POCTs and those in the pipeline.

**Results and discussions:**

The diagnostic accuracy of urethral discharge and genital ulcer disease syndromes is relatively adequate. Asymptomatic *Chlamydia trachomatis* (CT) and *Neisseria gonorrhoeae* (NG) infections limit the use of vaginal discharge and anorectal syndromes. The pooled diagnostic accuracy of vaginal syndromic case management for CT/NG is low, resulting in high numbers of overtreatment and missed treatment. The absolute effect of POCTs was reduced overtreatment and missed treatment. Findings of the reviews on syndromic case management underscored the need for low‐cost and accurate POCTs for the identification, first, of CT/NG, and, second, of *Mycoplasma genitalium* (MG) and *Trichomonas vaginalis* (TV) and NG and MG resistance/susceptibility testing. Near‐patient POCT molecular assays for CT/NG/TV are commercially available. The prices of these POCTs remain the barrier for uptake in resource‐constrained settings. This is driving the development of lower cost solutions.

**Conclusions:**

The WHO syndromic case management guidelines should be updated to raise the quality of STI management through the integration of laboratory tests. STI screening strategies are needed to address asymptomatic STIs. POCTs that are accurate, rapid, simple and affordable are urgently needed in resource‐constrained settings to support the uptake of aetiological diagnosis and treatment.

**Abbreviations**
AMR: Antimicrobial resistance
BV: bacterial vaginosis
CT: *Chlamydia trachomatis*
FDA: Food and Drug Administration
GUD: genital ulcer disease
HCP: health care provider
HIV: human immunodeficiency virus
HSV: herpes simplex virus
ICT: immunochromatographic tests
LFA: lateral flow assays
LMICs: low‐ and middle‐income countries
MG: *Mycoplasma genitalium*
MSM: men who have sex with men
NAAT: nucleic acid amplification tests
NG: *Neisseria gonorrhoeae*
OIA: optimal immunoassay
PCR: polymerase chain reaction
PID: pelvic inflammatory disease
POCT: point‐of‐care test
RDT: rapid diagnostic test
RPR: rapid plasma reagin
SDG: Sustainable Development Goal
SRH: sexual and reproductive health
STI: sexually transmitted infection
TP: *Treponema*
TPHA: *Treponema pallidum* haemagglutination
TV: *Trichomonas vaginalis*
WHO: World Health Organization

## 1 Introduction

Sexually transmitted infections (STIs) remain prevalent and a major burden of morbidity and mortality globally 1, impacting on quality of life, reproductive and child health, and national and individual economies. STIs also facilitate the sexual transmission of human immunodeficiency virus (HIV) 2, 3, 4. WHO reported an estimated 376 million infections of the four most common curable STIs (chlamydia, gonorrhoea, trichomoniasis and syphilis) occurred in 2016 5.

The global STI strategy, endorsed by the World Health Assembly in 2016 aims to end STIs as a public health threat by 2030 6.

Thus, appropriate STI diagnosis and treatment is crucial to prevent the transmission and sequelae of untreated infection 6, 7, 8. In resource‐constrained settings, aetiological diagnosis of STIs remains difficult due to limited access to laboratory diagnostics to guide appropriate treatment 8. Where facilities are available, tests results for people with suspected STIs take days or even weeks, making immediate treatment based on laboratory results unfeasible 8, 9.

To overcome limited access to aetiological diagnosis and treatment, syndromic case management was introduced by the World Health Organization (WHO) in 1984 and continues to be used as the standard of care by many countries, especially resource‐constrained ones 10. Syndromic management is based on the identification of consistent groups of symptoms and easily recognized signs (syndromes), and treatment that will deal with most, or the most serious, organisms responsible for producing the syndrome 11.

Syndromic management has been successful in reducing the prevalence of STIs over the years, such as chancroid and the incidence of male urethritis 12, 13, 14, but it has now reached its limits for several reasons. Most women with vaginal discharge do not have *Chlamydia trachomatis* (CT) and/or *Neisseria gonorrhoeae* (NG) 15, 16. Additionally, the cause of genital ulcer disease (GUD) syndrome has become less by chancroid or syphilis and more by herpes simplex virus (HSV) 14, 17. With the advent of molecular tests, it has become evident that many more infections exist asymptomatically in both men and women 18, 19 and that the diagnostic accuracy of STI syndromes is low 15, 16. In addition, the increasing rates of antimicrobial resistance (AMR) in NG and *Mycoplasma genitalium* (MG) with limited treatment options make it imperative that treatments are based on aetiological diagnosis 20, 21.

Point‐of‐care tests (POCTs) in accordance with the ASSURED criteria (affordable, sensitive, specific, user‐friendly, robust/rapid, equipment free and delivered to end users) are essential to address these challenges 22. While some POCTs exist, implementation barriers at the levels of device, patient, provider and health system make them unavailable in most resource‐constrained settings 23.

This paper reviews the diagnostic accuracy of syndromic case management, and the existing POCTs and those in the pipeline to detect STIs that could potentially be used in resource‐constrained settings.

## 2 Methods

Because of the challenges in diagnosing STIs in women, we prioritized updating the systematic review of studies from January 2001 to March 2015 and the meta‐analysis of the diagnostic accuracy of vaginal discharge by Zemouri et al. 24. We updated the search from January 2015 to September 2018 in OVID Medline and CENTRAL, and in EMBASE using the two strategies provided in Zemouri (2016). Studies that evaluated the diagnostic accuracy and validation of vaginal discharge flowchart compared to any laboratory diagnostic test were included. The search strategy and results are detailed in Supporting Information. In this review, all flowcharts (the index tests) had the entry point of women complaining of vaginal discharge followed by history taking, including risk assessment and genital inspection to verify the presence of vaginal discharge. Flowcharts were categorized as follows: flowchart 1 = history and risk assessment; flowchart 2 = history, risk assessment and speculum examination; flowchart 3 = history, risk assessment, speculum examination, and vaginal discharge samples for Gram staining and wet‐mount microscopy to diagnose the presence of budding yeast or psuedohyphae for *Candida albicans*, motile trichomonads for *Trichomonas vaginalis* (TV) and Amsel criteria for diagnosis of bacterial vaginosis (BV); and flowchart 4 = country‐adapted flowcharts with country‐specific risk factors or those not defined by the study methods. Four additional studies were added to the meta‐analysis 25, 26, 27, 28. We conducted a meta‐analysis by pooling of samples from all studies within different types of flowcharts. We calculated the pooled sensitivity and specificity for the different type of the flowcharts using the WINPEPI software (version 11.65, August 2016). If the study had presented the results separately for NG, CT, TV and BV, the study with the higher PPV was included in the meta‐analyses so as not to over represent any study.

Based on the diagnostic accuracy for CT/NG of different vaginal discharge flowcharts, we calculated absolute effects on important outcomes – true positive, false positive (resulting in overtreatment), true negative and false negative (resulting in incorrect or missed treatment) in different CT/NG prevalence settings (5%, 15%, 30%). We then calculated the absolute effects on the important outcomes in different CT/NG prevalence settings using rapid diagnostic tests (RDTs) with sensitivities of 60%, 70% and 80%, and specificity of 90%, to represent the ranges of sensitivity and the lowest acceptable specificity of the RDTs detailed in Table 5, and using a molecular assay with a sensitivity of 95% and specificity of 98%, that is, Xpert CT/NG on GeneXpert system 29, 30.

We searched the peer‐reviewed literature for previous systematic reviews, randomized controlled trials and non‐randomized studies from 2000 to 2018 on the diagnostic accuracy of syndromic management for vaginal and urethral discharge, genital ulcer and anorectal infections. We selected studies from searches of the PubMed and Medline databases. We chose articles that appropriately addressed the key issues and we did not apply eligibility criteria to include or exclude articles.

We conducted literature reviews on existing POCTs and those in the pipeline, on patient and healthcare provider (HCP) values and preferences, and on the costs and cost‐effectiveness of POCTs for STIs. We searched PubMed and Medline databases from 2000 to 2018. We used the search terms point of care, POC, POCT, rapid test, laboratory tests, laboratory diagnosis, aetiologic diagnosis and sexually transmitted infections/diseases. We searched reviews, editorials and systematic reviews for additional publications.

## 3 Results

### 3.1 Syndromic case management

Syndromic management for urethral discharge in men had sensitivities ranging from 84% to 95%. Treatment based on this syndrome is simple, inexpensive and cost‐effective 31, 32, 33, 34. Apart from CT/NG, aetiologies include MG and TV 35, 36, 37.

Genital HSV infection is the predominant cause of GUD that affects the outcome of syndromic management of GUD 14, 17, 38, 39, 40. In studies evaluating the GUD flowchart, only two in India made a distinction based on the appearance of the ulcer 39, 41, 42. Studies revealed the moderate sensitivity and low specificity of clinically differentiating herpetic (sensitivity, 74%; specificity, 33%) and non‐herpetic (sensitivity, 51%; specificity, 56%) 39, 41, 42.

The WHO simplified generic tool includes flowcharts for women with symptoms of vaginal discharge and/or lower abdominal pain. While the flowcharts for abdominal pain are relatively satisfactory 31, those for vaginal discharge have severe limitations. Systematic reviews and meta‐analyses of the syndromic approach to diagnose and treat cervical infections (CT/NG) revealed low accuracy, resulting in a high proportion of overtreatment, incorrect treatment and missed treatment 24, 31, 43, 44. In settings of low STI prevalence, endogenous vaginitis and BV, rather than CT/NG/MG, are the main causes of abnormal vaginal discharge 24, 31, 43, 44. Attempts to increase the sensitivity and specificity of the vaginal discharge flowchart for the diagnosis of cervical infection using situation‐specific risk assessment have not been successful 45, 46.

A review by Sloan et al. also revealed that syndromic management had low diagnostic accuracy for screening and case‐finding of CT/NG in women 43.

Based on our update of the systematic review and meta‐analysis by Zemouri et al. 24, the pooled sensitivity and specificity of the various flowcharts to diagnose vaginal infection (TV and BV) are summarized in Table 1.

**Table 1:** Pooled sensitivity and specificity of different syndromic flowcharts to diagnose vaginal infections (*Trichomonas vaginalis* and bacterial vaginosis) 24

Flowchart	Number of studies	Sensitivity, % (95% CI)	Specificity, % (95% CI)
1 (Risk assessment)	9	56.2 (54.5 to 57.9)	71.0 (69.4 to 72.6)
2 (+ speculum examination)	8	74.8 (74.0 to 75.6)	53.2 (52.5 to 54.0)
3 (+ Lab (WM, GS))	2	91.7 (89.2 to 94.2)	100 (99.9 to 100)
4 (Local adaptation)	5	53.1 (50.5 to 55.6)	85.8 (84.7 to 86.9)

Update of the systematic review and meta‐analysis by Zemouri et al. 24. CI, confidence interval; GS, Gram‐stained microscopy; WM, wet‐mount microscopy.

The pooled sensitivity and specificity of the various flowcharts to diagnose cervical infection due to CT/NG are summarized in Table 2.

**Table 2:** Pooled sensitivity and specificity of different syndromic flowcharts to diagnose *Chlamydia trachomatis* and *Neisseria gonorrhoeae* 24

Flowchart	Number of studies	Sensitivity, % (95% CI)	Specificity, % (95% CI)
1 (Risk assessment)	7	27.9 (24.7 to 31.1)	57.0 (56.1 to 58.0)
2 (+ speculum examination)	9	44.9 (42.2 to 47.7)	74.2 (73.3 to 75.1)
3 (+ Lab (WM, GS))	3	90.1 (85.8 to 94.4)	35.3 (33.4 to 37.1)
4 (Local adaptation)	7	83.92 (80.9 to 87.0)	45.3 (43.9 to 47.9)

Update of the systematic review and meta‐analysis by Zemouri et al. 24. CI, confidence interval; GS, Gram‐stained microscopy; WM, wet‐mount microscopy.

The absolute effect of different prevalence using the pooled sensitivities and specificities of the different vaginal discharge flowcharts reveal that the low diagnostic accuracy of vaginal syndromic case management results in high numbers of false positives (lower specificity), leading to overtreatment, and high numbers of false negatives (lower sensitivity), resulting in incorrect and missed treatment (Table 3). The absolute effects on outcomes in settings with different CT/NG prevalence using RDTs with sensitivities of 60%, 70%, 80% and a specificity of 90%, and with POCT molecular assay (sensitivity of 95% and specificity of 98%), reveal fewer false positives and false negatives and more true positives compared with syndromic case management (Table 4).

**Table 3:** Absolute effects on outcomes using the diagnostic accuracy of different vaginal syndromic flowcharts to diagnose *Chlamydia trachomatis* and *Neisseria gonorrhoeae* in settings with different prevalence

Cervical infections				Prevalence (per 1000)		
Sensitivity	Specificity	Flowchart	Outcomes	50	150	300
0.28	0.57	Flowchart 1	TP	14	42	84
			FN – missed treatment	36	108	216
			TN	542	485	399
			FP – overtreatment	409	366	301
0.45	0.74	Flowchart 2	TP	22	67	135
			FN – missed treatment	28	83	165
			TN	705	631	519
			FP – overtreatment	245	219	181
0.90	0.35	Flowchart 3	TP	45	135	270
			FN – missed treatment	5	15	30
			TN	335	300	247
			FP – overtreatment	615	550	453
0.84	0.45	Flowchart 4	TP	42	126	252
			FN – missed treatment	8	24	48
			TN	430	385	317
			FP – overtreatment	520	465	383

FP, false positive; FN, false negative; TN, true negative; TP, true positive.

**Table 4:** Absolute effects on outcomes using the diagnostic accuracy of rapid diagnostic tests and molecular point‐of‐care tests to diagnose *Chlamydia trachomatis* and *Neisseria gonorrhoeae* in settings with different prevalence

Cervical Infections				Prevalence (per 1000)		
Sensitivity	Specificity	Test	Outcome	50	150	300
0.6	0.9	RDT 1	TP	30	90	180
			FN – missed treatment	20	60	120
			TN	855	765	630
			FP – overtreatment	95	85	70
0.7	0.9	RDT 2	TP	35	105	210
			FN – missed treatment	15	45	90
			TN	855	765	630
			FP – overtreatment	95	85	70
0.8	0.9	RDT 3	TP	40	120	240
			FN – missed treatment	10	30	60
			TN	855	765	630
			FP – over overtreatment	95	85	70
0. 95	0.98	Molecular POCT assay	TP	47	142	285
			FN – missed treatment	3	8	15
			TN	931	833	686
			FP – overtreatment	19	17	14

FP, false positive; FN, false negative; POCT, point‐of‐care test; RDT, rapid diagnostic test; TN, true negative; TP, true positive.

The flowchart for syndromic management of anorectal infections intends to treat CT/NG rather than being solely based on symptoms and signs 47, 48. This is similar to treating cervical infection (CT/NG) in the vaginal discharge flowchart. The limitations are thus similar with rectal infections, where the majority are asymptomatic 19, 49. In a small study in Kenya, one in five men with an anorectal CT/NG reported rectal pain 50; in Côte d'Ivoire, more than half of the men in the study reported anorectal symptoms in the past 12 months 51; in Germany, 12% of 2247 men who have sex with men (MSM) had anorectal CT/NG, and only 12% of these had local symptoms, and 91% of both rectal and pharyngeal CT/NG would have been missed if only symptomatic men had been tested 52.

Unprotected anal sex is the entry point to the flowchart for anorectal infections. While it is recommended that carefully worded questions can be used to elicit anal sex in sub‐Saharan Africa 53, it is unlikely that many MSM will respond appropriately, especially where homosexuality is illegal 54. A substantial proportion of potential patients is thereby excluded from the flowchart.

### 3.2 Aetiological diagnosis of STIs

Nucleic acid amplification tests (NAATs) are the gold standard for the diagnosis of STIs in high‐income settings, and most have a sensitivity and specificity ranging from 95% to 99% 6. Several laboratory tests and procedures for specific STIs are elaborated in a WHO manual 55. Most of the recommended highly sensitive and specific NAATs require resources, training, laboratory infrastructure, longer time for results, and are expensive, thus making them inaccessible for many resource‐constrained settings 23.

### 3.3 Point‐of‐care tests for common STIs

#### 3.3.1 Syphilis

Syphilis prevalence is increasing in many countries 56, 57, 58. Untreated syphilis in pregnant women is a major cause of foetal death and congenital infection 59. WHO recommends syphilis screening for pregnant women, MSM and sex workers, and RDTs have increased screening uptake 48, 60.

There are several syphilis RDTs – rapid POCTs, that is – for screening (e.g. Determine, SD Syphilis 3.0, Syphicheck, Syphilis Rapid Test and Visitect). Most of these tests use whole blood, plasma or serum and can be performed between five and thirty minutes. Based on a meta‐analysis by Jafari et al., sensitivity ranges from 75% to 99% and specificity from 92% to 99% compared with *Treponema pallidum* haemagglutination (TPHA) and *Treponema pallidum* particle agglutination tests 61.

The main challenge with most syphilis RDTs, detecting only “specific” treponemal (TP) antibodies, is the inability to differentiate active from previously treated infection. To reduce overtreatment, especially in high‐prevalence populations (>5%), an initial RDT is performed and, if positive, this is followed by a rapid plasma reagin (RPR) test, which detects non‐TP antibodies, indicating an active infection. If the RPR test is reactive, treatment for syphilis is provided 60, 62. However, the uptake of the sequential RPR test is unknown in many resource‐constrained settings. To overcome the challenges with using RPR as a sequential test, a combination RDT screen‐and‐confirm assay has been developed to detect both TP and non‐TP antibodies. A meta‐analysis by Marks et al. showed that the sensitivity was higher in patients with higher RPR titre (≥1:16) for both the TP (98.2% vs. 90.1%, *p* < 0.0001) and the non‐TP component (98.2% vs. 80.6%, *p* < 0.0001). Overall agreement with TPHA was 85.2% (84.4% to 86.1%). Agreement was highest for high‐titre active infection, and lowest for past infection 62.

HIV testing has been scaled up in most countries, while syphilis screening lags behind. Implementing a combination test of HIV and syphilis will increase syphilis screening coverage, contributing to eliminating mother‐to‐child transmission of HIV and syphilis 59. A review by Gliddon et al. 63 showed that the diagnostic accuracy of the HIV component of the dual test ranged from 94% to 99% sensitivity and from 92% to 100% specificity. The syphilis diagnostic accuracy ranged from 47% to 96% sensitivity and 90% to 100% specificity. The lowest sensitivity reflected the low diagnostic performance of the test using whole blood. Sensitivity was higher for patients with non‐treponemal titres of >1:4, indicating that the syphilis test is more likely to detect active, transmissible infections versus old treated infection. The dual RDT was more cost effective than single RDTs and prevented more adverse outcomes of pregnancy. Qualitative data indicated that dual tests were acceptable in terms of turnaround time, cost and a single finger prick 63.

#### 3.3.2 *Chlamydia trachomatis* and *Neisseria gonorrhoeae*

CT infection remains the most prevalent bacterial STI 5 and is often asymptomatic 64, 65. About 10% to 40% of patients are co‐infected with NG 37, 65, 66, 67, 68. Appropriate laboratory diagnostic tests are essential to screen for asymptomatic CT. Gonorrhoea is the second most prevalent reported bacterial STI 5 and usually asymptomatic in women 16, 18. Because of an increase in NG AMR to the currently recommended treatment for gonorrhoea, laboratory diagnosis is essential 20, 21. If CT/NG infections remain untreated, they can result in infertility, adverse outcomes of pregnancy, newborn infections and increased risk of HIV transmission 6, 69.

CT antigen detection POCTs are available. As described in a recent systematic review by Kelly et al. 70, these lateral flow assays (LFA)/immunochromatographic tests (ICT) include ACON chlamydia, aQcare Chlamydia TRF kit, BioRapid Chlamydia Ag test, Chlamydia Rapid Test SAS, Clearview Chlamydia, and QuickVue. The specificity of these rapid POCTs was high across all specimen types (97% to 100%); however, the sensitivities were low (37% for vaginal swabs, 53% for endocervical swabs and 63% for urine). The new aQcare Chlamydia TRF kit, a fluorescent nanoparticle‐based LFA, was the best performing POCT, with sensitivities and specificities comparable to POCT NAATs 70.

There have been fewer POCTs developed for gonorrhoea, and many have been validated only by the manufacturer and are not currently commercially available. The diagnostic sensitivities of these tests are generally lower than of the CT LFAs/ICTs (Table 5).

**Table 5:** Rapid point‐of‐care tests for diagnosis of *Neisseria gonorrhoeae*

Test Name	Manufacturer	Commercially available	Sensitivity (%)	Specificity (%)	Reference Test	Sample type
ACON CT/NG Duo 71	ACON	No	12.5	99.8	NAAT (Roche Cobas)	Endocervical swab
ACON NG 71	ACON	No	Not quantified	97.2	NAAT (Roche Cobas)	Endocervical swab
BioStar Optical ImmunoAssay 72	Thermo Biostar	No	100^a^	93	NAAT (Hologic Aptima)	Urine (males)
			30 to 60	60 to 90	Culture	Endocervical swab
GC‐Check 73	PATH	No	70	97.2	NAAT (Roche Amplicor)	Endocervical swab
			54.1	98.2	NAAT (Roche Amplicor)	Vaginal swab
OneStep Gonorrhea RapidCard Insta Test 74	Cortez Diagnostics	No	64 to 94	67 to 97	Culture	Endocervical swab
			61 to 91	67 to 97	Culture	Urethral swab (male)
GC RapidResponse	BTNX	Yes	64 to 94	67 to 97	Culture	Endocervical swab
			61 to 91	67 to 97	Culture	Urethral swab (male)
GC One‐step test	Novamed	Yes	68 to 98	68 to 98	Culture	Vaginal swab/Urethral swab (male)

NAAT, nucleic acid amplification test.

a Very limited evaluation, including only five *N. gonorrhoeae*‐positive clinical specimens from males with symptomatic urethritis.

The performance of some NG POCTs was evaluated only against culture, and not the more accurate NAATs (gold‐standard test), and only symptomatic patients were included in the evaluation. No gonorrhoea POCT has been evaluated for extragenital sites. Rapid POCTs (LFAs, ICTs and OIAs) take five to seven steps, but have turnaround times of only 25 to 40 minutes, making them suitable for primary care settings 75.

The near‐patient Xpert CT/NG (real‐time NAAT) on the GeneXpert instruments is approved by the United States Food and Drug Administration (FDA). The diagnostic accuracy from self‐collected vaginal swabs, cervical swabs and urine range from 95% to 98%, with specificities ranging from 99.4% to 99.9%. The sensitivity and specificity of this assay for rectal swabs are 86.0% and 99.2% respectively 30, 76.

The Xpert CT/NG takes three steps and 90 minutes, and requires equipment (GeneXpert), steady electricity, calibration, a temperature‐controlled environment 73. Several studies have shown that this can be used in settings with basic laboratory infrastructure. The utility of GeneXpert has been evaluated in remote populations such as an aboriginal community in Australia 77; in routine antenatal care in Papua New Guinea (with STI rates by GeneXpert of CT 20%, NG 11.2% and TV 37.6%) 78; in HIV‐infected pregnant women in South Africa (40.2% with STIs) 79. Another utility study in South Africa in HIV‐negative women presenting for STI care or with symptoms (CT 18.4%, NG 5.2%, TV 3%) resulted in STI testing of symptomatic and asymptomatic women and the same‐day treatment, with expedited partner treatment and reduced reinfection after six months 80. A study in Rwanda has shown that integrating POCTs for BV, TV (OSOM) and CT/NG (GeneXpert) in women with urogenital symptoms and increased risk of STIs has improved diagnostic accuracy, with moderate sensitivity and high specificity for CT/NG/TV compared with using syndromic management, and has remarkably reduced overtreatment 81.

Several platforms and assays are being developed to be more portable, easier to operate, used at the point of care and giving rapid turnaround times for results, with accuracy similar to that of laboratory‐based NAATs, such as the GeneXpert Omni, Alere – i platform, RT CPA CT Test, Atlas Genetics io Platform 82 and the Truelab Real Time micro PCR system 76, 83, 84.

POCTs that are inexpensive, rapid and fulfil the ASSURED criteria are under development. These molecular assays include: the microwave‐accelerated metal‐enhanced fluorescence test, which needs to be simplified and standardized for basic laboratories 84; a low‐cost NAAT called MobiNAAT, which uses a portable device where results are analysed in an automated smartphone diagnostic 83, 84; a POCT paper‐fluidic platform to diagnose gonorrhoea that is a highly sensitive molecular assay with visual lateral flow detection and an 80‐minute run time 86; and a rapid multiplex microfluidic CT PCR‐based POCT comparable to laboratory‐based NAATs 87, 88. A 15‐minute run‐time recombinase polymerase amplification‐based prototype POCT (TwistDx) for CT/NG has been reported to be comparable to laboratory‐based NAAT 89. Improvement of the sensitivity of some LFAs for CT has been described 90.

Some companies are working on antigen‐ or protein‐based detection of AMR in NG (i.e. LFA‐type tests). However, this is very early work, and development and commercial pathways are unclear, as are timelines. There are several well‐characterized molecular AMR determinants that can be used for effective prediction of AMR in NG, particularly for ciprofloxacin, but less adequate prediction of resistance to azithromycin, cefixime and ceftriaxone 91, 92.

#### 3.3.3 Trichomonas vaginalis

TV is the most prevalent curable STI globally and is a major cause of vaginal discharge as well as recurrent urethral discharge in men 5, 16, 24, 36, 37. Wet‐mount microscopy is the most common method of diagnosing TV, because it is cheap and rapid but with a sensitivity from 44% to 68% 93. TV culture (e.g. InPouch TV) has a sensitivity ranging from 44% to 75% for women 93. Gaydos et al. conducted a systematic review of TV diagnostic tests 94. Based on this review, the rapid POCT OSOM lateral flow test has a sensitivity ranging from 83% to 86%. The AmpliVue and Solana tests are near‐patient NAATs, requiring a small piece of equipment, with a sensitivity of 90.7% for AmpliVue, and 98.6% for Solana test for vaginal swabs and 100% for urine specimens. In addition, the near‐patient Xpert TV assay on GeneXpert is now available with around 96% sensitivity for vaginal swabs and 97% sensitivity for urine samples. These new molecular diagnostic assays have a high diagnostic accuracy with rapid turnaround times, and enable the detection of TV in urine in men 94.

#### 3.3.4 Healthcare provider perception of point‐of‐care tests

Qualitative studies conducted by Hsieh et al. 95 to assess the requirements placed on HCPs by POCTs revealed that an ideal POCT should be like a pregnancy test that can be purchased over the counter for home use. It should be simple to use and interpret and take around 20 minutes to run and release the result. Moreover, the turnaround time should coincide with the time spent for the patient–client interaction. Most HCPs indicate that the accuracy of the test should be the same as that of a laboratory‐based NAAT 95.

HCPs have expressed confidence in the POCT NAAT results, and treating patients on this basis 96. They mentioned that POCTs provide an opportunity for targeted patient treatment, immediate partner notification and reduced follow‐up effort 95. However, the main barriers indicated were the long waiting time, the time consumed in the documentation process, sample collection, inadequate training and the limited availability of POCTs due to a high unit cost per test 95, 96, 97.

## 4 Discussion

The provision of effective services to symptomatic and ideally also asymptomatic STI patients and their partners should be among the top priorities of an STI control programme. Symptomatic STI patients may be aware that they are infected and are more likely to seek care. Thus, syndromic management provides an entry point for STI management and control. However, there are clearly limitations to the syndromic approach for the management of STIs, the likely impact on the control of STIs and the link with AMR 4, 9, 80.

While urethral discharge has relatively adequate diagnostic accuracy, treatment has been limited to CT/NG. It is also critical to address asymptomatic CT/NG and to assess the aetiologies of persistent urethral discharge, including MG and TV, as well as the treatment failures due to AMR in NG and MG.

Previous syndromic management has not considered MG as an important aetiological agent of urethral and vaginal discharge and pelvic inflammatory disease (PID). MG frequently causes non‐gonococcal urethritis (NGU) and non‐chlamydial‐NGU in men and is associated with vaginal discharge and PID in women 98, 99, 100. The high‐level of AMR in MG and the lack of effective first‐line treatment 21 further complicate the inclusion of MG in syndromic management flowcharts.

Most NG, and especially CT and MG, cervical infections in women are subclinical or asymptomatic so there would be no syndromic presentation 15, 18, 24, 31, 43. The syndromic approach has never been intended as a tool for case finding or for screening asymptomatic patients 43 and, predictably, this misuse of the approach has led to disappointments.

Based on the available evidence, vaginal discharge syndrome has adequate diagnostic accuracy to detect vaginal infections (TV and BV) (Table 1), but has very poor diagnostic accuracy for cervical infection (CT/NG) (Table 2). The absolute effect for diagnosing cervical infections (Table 3) is a high number of false‐positive CT/NG cases, resulting in a higher number of individuals being overtreated with extended‐spectrum cephalosporins and azithromycin/doxycycline. This can lead to adverse reactions, can facilitate AMR and can create the social and individual effects of falsely being diagnosed with an STI. There is also a high rate of false negatives, resulting in missed treatment, which can facilitate further transmission and severe complications and/or sequelae. On the other hand, RDTs and POCTs (Table 4) can reduce overtreatment and missed treatment by adapting antibiotic prescriptions according to test results, and can facilitate partner notification 80.

Patients with vaginal and urethral discharge syndromes are mostly seen in primary care settings, which do not have accessible diagnostics to confirm either CT/NG/MG/TV. Although one FDA‐approved near‐patient (POCT) molecular assay (Xpert CT/NG) is available to distinguish between CT and NG, the cost and other limitations 75, 101 remain prohibitive for use in primary care.

The severity of symptoms associated with various STI pathogens and the anatomical sites infected greatly influence treatment‐seeking behaviour 102, 103. Men with NG are frequently symptomatic 32, 33, 34 whereas women with CT, NG and MG are frequently asymptomatic 15, 18, 24. Many syphilis cases occur without symptoms 59, as do many anal CT/NG infections 48, 49. Different interventions are thus necessary. Prompt access to effective services for symptomatic infections remains an important approach (syndromic management and integration of POCT), while screening and treatment for syphilis and chlamydial infection, and screening of high‐risk populations for CT/NG, are needed.

AMR to the first‐line NG treatment regimen of ceftriaxone plus azithromycin, and AMR in MG to azithromycin (first‐line) and moxifloxacin (second‐line), has now been reported 20, 21, 99, 100. Because of the low diagnostic accuracy of the syndromic approach to diagnose CT/NG, there is significant overuse of these therapies, which could contribute to AMR emergence. A diagnostic‐based antibiotic stewardship strategy is urgently needed. A near‐term solution requires a rapid, easy‐to‐use, low‐cost assay to distinguish between CT, NG and MG. Additionally, a rapid, easy‐to‐use, low‐cost assay to determine susceptibility to currently available antibiotics in confirmed NG and possibly MG‐positive infections is needed. A longer‐term solution will be to incorporate these tests into one assay and to distinguish between multiple STIs as well as detect resistance/susceptibility.

This review highlights the need to integrate currently available laboratory‐based diagnostics and POCTs within syndromic case management to decrease overtreatment and missed treatment as well as to contribute to the conservation of NG treatment. A recent study by Verwijs et al. 81 has shown that integrating POCT (CT, NG, TV) in women with urogenital symptoms and for screening resulted in the reduction of NG and CT by half, and of TV by 42% 81.

Laboratory diagnostics will also be essential for implementing STI screening strategies. The unit cost per test can be higher compared with treatment costs, which often remains the major concern of national programmes in investing in laboratory diagnosis. However, the cost savings obtained from the rapid delivery of results, reduction of patient follow‐up, facility cost, decreased complications and onward transmission are often overlooked 104. For example, based on modelling by Vickerman et al., a POCT with a 70% to 80% sensitivity, 95% specificity and a cost of about US$1‐2 would be a cost‐effective strategy for substantially reducing the impact in HIV transmission and the degree of inappropriate and missed treatment from using syndromic management to diagnose CT/NG in high prevalence settings 105. The cost‐effectiveness of multiplex POCTs (CT, NG, MG, TV) has been demonstrated in a separate modelling study 106.

A cost‐effectiveness analysis has shown that a NG NAAT screening of women between 15 and 29 years of age can prevent 1247 cases of PID and save US$177 per patient compared with no screening, while using a potential POCT with about 75% sensitivity can prevent additional PID 107.

Supplementing the laboratory‐based NAATs for CT/NG with POCTs NAAT could be cost‐saving and patients could benefit from accurate diagnosis, and immediate and appropriate treatment. POCTs can reduce overtreatment and eliminate the need for presumptive treatment 108, 109. A promising CT POCT (with a sensitivity of 92.7%, with 47% of women willing to wait and a test cost of US$33) will likely be cost‐effective compared with a traditional NAAT, which could save US$28 in total and avert more PID cases 110.

Modelling the impact of a rapid testing service showed that it could reduce the mean time to treatment notification from eight days to less than a day, and avert more CT/NG transmission. Additionally, there is an annual saving in the number of partner attendances 111.

POCT with AMR detection has shown that there is an additional cost for this POCT, but its use could reduce the cost from follow‐up visits and could allow for the use of older and cheaper drugs, such as ciprofloxacin and, more importantly, conserve the current last‐resort options of ceftriaxone and azithromycin 112.

Test cost is a significant factor in the use of available NAATs and the development and utility of POCTs. Although cost‐effective, the unit cost per test of a NAATs ranges from US$14 to US$30 per sample, which is often unaffordable in resource‐constrained settings 101, 113. There are urgent needs to develop low‐cost, simple and rapid POCTs for CT/NG/MG/TV with appropriate performance (accuracy and operational characteristics) to support uptake and widescale use in community settings. An acceptable diagnostic accuracy that will allow the development of more affordable POCTs than are currently available needs to be stipulated. Several compromises may have to be made with the ASSURED criteria 22. For instance, a cheap assay that has a sensitivity of about 80% and a specificity of at least 90% (Table 4), similar to a syphilis RDT, could be widely used and very valuable if it is affordable and integrated within a vaginal discharge flowchart 114, 115. These potential RDTs/POCTs would be more widely used in primary care and resource‐constrained settings and could possibly have a greater public health impact 101, 109, 114, 115.

The potential use of molecular diagnostic assays in resource‐constrained settings is driving the development of lower‐cost solutions. Several new industry players have entered, or are entering the development space; however, these tests, previously mentioned 76, 85, 86, 87, 88, 89, 90, are mostly in the early stages of development – and it remains to be seen how these assays perform, and what the global access pricing strategies will be.

The development of POCTs will need to ensure access and uptake at the primary health care level. Self‐sampling (e.g. urine, and high vaginal swabs) has shown to increase POCT use and is thus an important consideration in POCT development 116, 117. Self‐testing and sampling have increased screening uptake, but innovative treatment services to avoid ineffective and inappropriate treatment should be explored 118, 119, 120. POCT implementation should consider integration within the STI management pathways, including patient flow, immediate treatment, partner management and retesting 121, and the existing health systems 122.

## 5 Conclusions

In the present review, the available evidence on the effectiveness and challenges of syndromic case management further underscores the need to scale up existing STI diagnostics and the development of POCTs for, first, the identification of CT/NG, but ideally also MG and TV, as well as NG and MG AMR in vaginal, urethral and anorectal discharge.

One of the biggest challenges in STI control is that most cases are asymptomatic or have unrecognized symptoms 6, 7, 8, 9. POCTs will increase the uptake of STI screening in vulnerable populations that are at highest risk and will have an impact on detection and treatment. 121, 123, 124, 125, 126.

Although near‐patient NAAT for CT/NG/TV is commercially available, the cost and other limitations remain prohibitive for use, particularly but not exclusively in resource‐constrained settings 9, 20, 101.

POCTs that are simple and affordable are essential in STI control and are urgently needed in resource‐constrained settings. The development and implementation of POCTs will require innovative financing approaches and implementation strategies, and the strengthening of laboratory capacity. Although several POCTs for CT/NG are in the pipeline, the development of affordable POCTs will take several more years. Syndromic management of symptomatic STIs will remain essential in resource‐constrained settings. At the interim, guidelines should be updated to improve the standard of care and to explore the utility of available POCTs and near‐patient NAATs to improve STI diagnosis and screening. Laboratory and clinical validation studies and cost‐effectiveness analyses of integrating POCTs into current syndromic case management, and of screening strategies, are urgently needed to inform guidelines and national policies.

The limitation of the syndromic approach, the availability of molecular assays and the ongoing development of POCTs call for global action to increase the access and affordability of the aetiologically based diagnosis of STIs in resource‐constrained settings to improve patient management, and reduce STI transmission and the emergence of drug resistance. Finally, global initiatives are needed to make current near‐patient NAATs more affordable through subsidized cost and bulk procurement.

## Competing interests

The authors declare no conflicts of interest.

## Authors’ contributions

TW drafted the review and all authors contributed. All authors contributed in the final review of the manuscript. NS designed and conducted the search and data extraction for the vaginal discharge syndrome systematic review and meta‐analysis.

## Supporting information


**Data S1.** Updated systematic review of vaginal discharge.
**Figure S1.** PRISMA flow diagram
