# Supplementary material for: Diagnosing sexually transmitted infections in resource‐constrained settings: challenges and ways forward
Source: J Int AIDS Soc. 2019 Aug 30;22(Suppl Suppl 6):e25343. doi: 10.1002/jia2.25343 (PMC6715950; doi:10.1002/jia2.25343)
Supplement: Supplementary file 1 — Data S1. Updated systematic review of vaginal discharge. Figure S1. PRISMA flow diagram [file JIA2-22-e25343-s001.docx]

# Additional file 1: Updated systematic review of vaginal discharge

**Methodologies**

We performed an update of the systematic review for vaginal discharge by Zemouri 2016.

***Electronic search and study selection***

The original review searched various databases up to March 2015. We updated the search from January 2015 to September 2018 in OVID Medline and CENTRAL, and in EMBASE using the two strategies provided in Zemouri (2016).

Studies that evaluated the diagnostic accuracy and validation of vaginal discharge flowchart compared to any laboratory diagnostic test were included. Studies that did not distinguish between cervical infections [caused by *Neisseria gonorrhoeae* (NG) and *Chlamydia trachomatis* (CT)] and vaginal infections [caused by *Trichomonas vaginalis* (TV) and *Bacterial vaginosis* (BV)] were not included in the final review. Studies that presented data on sensitivity, specificity, positive predictive values (PPV), negative predictive value (NPV) or that provided data from which these parameters could be calculated using two by two tables were included. We excluded studies published in languages other than English, French, Spanish and Dutch. Case reports and letter to editor were excluded. Two investigators (AMB and SD) assessed the studies for relevance, title, abstract, and content and applied the inclusion criteria to the full text articles. In case of disagreement between the reviewers, a discussion followed in order to reach consensus, otherwise the principal investigator (NS) was consulted.

***Index tests***

We followed Zemouri’s categorization of flowcharts (the index tests), as such:

- Flowchart 1 = history and risk assessment;
- Flowchart 2 = history, risk assessment and speculum examination;
- Flowchart 3 = history, risk assessment, speculum examination, and vaginal discharge samples for Gram staining and microscopy;
- Flowchart 4 = country adapted flowcharts or those not defined by the study method.

***Statistical analysis***

We conducted a meta-analysis by pooling of samples from all studies within different types of flowcharts. We calculated the pooled sensitivity and specificity for the different type of the flowcharts using the WINPEPI software (version 11.65, August 2016). If the study had presented the results separately for NG, CT, TV and BV, the study with the higher PPV was included in the meta-analyses so as not to over represent any study.

***Risk of bias of included studies***

We assessed the risk of bias of the different studies using the QUADAS-2 assessment tool. We graded as high, low or unclear the risk of bias for patient selection, index test, reference standard, flow and timing.

**Results**

***Study selection***

The two detailed search strategies are below with hits:

Database: EBM Reviews - Cochrane Central Register of Controlled Trials <August 2018>, Ovid MEDLINE(R) and Epub Ahead of Print, In-Process & Other Non-Indexed Citations and Daily <2014 to September 20, 2018>
--------------------------------------------------------------------------------
1     exp Vaginal Discharge/ (226)
2     vaginal discharge.ti,ab. (1040)
3     vaginal discharges.ti,ab. (40)
4     leukorrhea.mp. (134)
5     cervical discharge.mp. (28)
6     Cervix Uteri/ (3998)
7     cervical discharges.mp. (2)
8     vaginal.mp. (36974)
9     discharge.mp. (85265)
10     8 and 9 (1957)
11     vagina.mp. (10943)
12     9 and 11 (645)
13     cervix.mp. (15547)
14     9 and 13 (297)
15     vaginal secretion.mp. (124)
16     Software Design/ (916)
17     flowcharts.mp. (110)
18     Flowchart.mp. (466)
19     algorithm.mp. (84700)
20     algorithms.mp. (94011)
21     flow charts.mp. (112)
22     flow chart.mp. (403)
23     clinical pathway.mp. (981)
24     clinical pathways.mp. (804)
25     risk assessment.mp. (97092)
26     syndromically.mp. (21)
27     syndromic.mp. (4848)
28     signs.mp. (97431)
29     symptoms.mp. (365438)
30     symptom.mp. (111199)
31     sign decision tree.mp. (0)
32     syndromic approach.mp. (82)
33     syndromic diagnosis.mp. (95)
34     syndromic management.mp. (123)
35     syndromic approaches.mp. (4)
36     (Software Design or flowcharts or Flowchart or algorithm or algorithms or flow charts or flow chart or clinical pathway or clinical pathways or risk assessment or syndromically or syndromic or signs or symptoms or symptom or sign decision tree or syndromic approach or syndromic diagnosis or syndromic management or syndromic approaches).mp. (717848)
37     discharges.mp. (9117)
38     13 and 37 (7)
39     1 or 2 or 3 or 4 or 5 or 6 or 7 or 10 or 12 or 14 or 15 or 38 (6324)
40     36 and 39 (1053)
41     limit 40 to yr="2015 -Current" (546)
42     remove duplicates from 41 (513)

Database: Embase <1996 to 2018 September 20>

--------------------------------------------------------------------------------

1 exp vagina discharge/ (6641)

2 fluor vaginalis.mp. (29)

3 genital fluor.mp. (1)

4 vagina fluid.mp. (4)

5 vagina fluor.mp. (0)

6 vaginal discharge.mp. (3111)

7 vaginal fluid.mp. (965)

8 vaginal fluor.mp. (5)

9 leukorrhea.mp. (697)

10 exp leukorrhea/ (661)

11 fluor albus.mp. (4)

12 cervical discharges.mp. (2)

13 vaginal.mp. (108743)

14 discharge.mp. (264824)

15 13 and 14 (8193)

16 vagina.mp. (65309)

17 14 and 16 (8017)

18 cervix.mp. (111422)

19 14 and 18 (2082)

20 discharges.mp. (28343)

21 18 and 20 (53)

22 vaginal secretion.mp. (740)

23 exp uterine cervix/ (13159)

24 secretion.mp. (319639)

25 discharge.mp. (264824)

26 discharges.mp. (28343)

27 secretions.mp. (22068)

28 24 or 25 or 26 or 27 (609769)

29 23 and 28 (1038)

30 exp algorithm/ (247451)

31 flowcharts.mp. (353)

32 Flowchart.mp. (1243)

33 algorithm.mp. (308870)

34 algorithms.mp. (89527)

35 flow charts.mp. (414)

36 flow chart.mp. (1378)

37 clinical pathway.mp. (8931)

38 clinical pathways.mp. (2579)

39 risk assessment.mp. (469668)

40 syndromically.mp. (50)

41 syndromic.mp. (12598)

42 signs.ti,ab. (289594)

43 symptoms.mp. (968757)

44 symptom.mp. (428354)

45 sign.ti,ab. (83781)

46 decision tree.mp. (12424)

47 decision trees.mp. (1932)

48 syndromic approach.mp. (258)

49 syndromic diagnosis.mp. (343)

50 syndromic management.mp. (368)

51 syndromic approaches.mp. (12)

52 (algorithm or flowcharts or Flowchart or algorithm or algorithms or flow charts or flow chart or clinical pathway or clinical pathways or risk assessment or syndromically or syndromic or signs or symptoms or symptom or sign or decision tree or decision trees or syndromic approach or syndromic diagnosis or syndromic management or syndromic approaches).mp. (2208740)

53 1 or 2 or 3 or 4 or 5 or 6 or 7 or 8 or 9 or 10 or 11 or 12 or 15 or 17 or 19 or 21 or 22 or 29 (13368)

54 52 and 53 (3697)

1. limit 54 to yr="2015 -Current" (1056)

In total, we found 1,569 citations (duplicates included). After removal of 311 duplicates, there were 1,258 citations for title and abstract screening. After excluding 1,086 studies that were not relevant, we obtained and screened 173 full text articles. We included four studies in the updated review (Bannaheke[1], Barry [2], Molaei [3], Vallely [4]). See PRISMA diagram below.

Therefore, in addition to the 16 studies from the previous review, the updated systematic review now includes 20 studies.

References:

1. Zemouri, C., Wi, T. E., Kiarie, J., Seuc, A., Mogasale, V., Latif, A., Broutet, N. The performance of the vaginal discharge syndromic management in treating vaginal and cervical Infection: A systematic review and meta-analysis. *PloS One.* 2016 Oct; 11(10), e0163365. doi:10.1371/journal.pone.0163365
2. Banneheke H, Fernandopulle R, Gunasekara U, Barua A, Fernando N, Wickremasinghe R. Can trichomonas immunochromatographic test increase the validity and reliability of WHO syndromic algorithm for vaginal discharge as a screening tool for trichomoniasis? *Ann Trop Med Public Health.* 2016; 9:43-7.
3. Barry MS, Ba Diallo A, Diadhiou M, Mall I, Gassama O, Ndiaye Gueye MD, Covi-Alavo S, Gawa E, Ndao Fall A, Gaye Diallo A, Moreau JC. Accuracy of syndromic management in targeting vaginal and cervical infections among symptomatic women of reproductive age attending primary care clinics in Dakar, Senegal. *Trop Med Internat Health.* 2018 May; 23(5): 541-548. doi:10.1111/tmi.13046
4. Molaei B, Mohmmadian F, Tadayon P, Gholami H, Kiani M, Rashtchi V. Comparative evaluation of accuracy and compatibility level of different diagnostic methods for bacterial vaginosis. *Kuwait Medical Journal.* 2018 Jun; 50(2): 205-212.
5. Vallely, L. M., et al. Performance of syndromic management for the detection and treatment of genital Chlamydia trachomatis, Neisseria gonorrhoeae and Trichomonas vaginalis among women attending antenatal, well woman and sexual health clinics in Papua New Guinea: a cross-sectional study." *BMJ Open.* 2017 Dec; 7 (12): e018630. doi:10.1136/bmjopen-2017-018630

**PRISMA Flow Diagram**

Records identified through database searching
(n = 1,569)

Records after duplicates removed
(n = 1,258)

Records excluded
(n = 1,086)

Records screened
(n = 1,258)

Full-text articles excluded, with reasons
(n = 171)

Full-text articles assessed for eligibility
(n = 173*)

Studies included in qualitative synthesis
(n = 4)

*Screening the record ‘Anonymous (2017). "IDSOG Abstracts 2017." American Journal of Obstetrics and Gynecology. Conference: 44th Annual Scientific Meeting Infectious Disease Society for Obstetrics and Gynecology, IDSOG 217(6)’ identified 2 articles requiring assessment for eligibility.
